# Targeted Alpha Therapy in mCRPC (Metastatic Castration-Resistant Prostate Cancer) Patients: Predictive Dosimetry and Toxicity Modeling of ^225^Ac-PSMA (Prostate-Specific Membrane Antigen)

**DOI:** 10.3389/fonc.2020.531660

**Published:** 2020-11-05

**Authors:** Maria Luisa Belli, Anna Sarnelli, Emilio Mezzenga, Francesco Cesarini, Paola Caroli, Valentina Di Iorio, Lidia Strigari, Marta Cremonesi, Antonino Romeo, Silvia Nicolini, Federica Matteucci, Stefano Severi, Giovanni Paganelli

**Affiliations:** ^1^Medical Physics Unit, Istituto Scientifico Romagnolo per lo Studio e la Cura dei Tumori (IRST) IRCCS, Meldola, Italy; ^2^Nuclear Medicine Unit, Istituto Scientifico Romagnolo per lo Studio e la Cura dei Tumori (IRST) IRCCS, Meldola, Italy; ^3^Oncology Pharmacy, Istituto Scientifico Romagnolo per lo Studio e la Cura dei Tumori (IRST) IRCCS, Meldola, Italy; ^4^Medical Physics Unit, Policlinico S. Orsola-Malpighi, Bologna, Italy; ^5^Radiation Research Unit, European Institute of Oncology (IEO) IRCCS, Milano, Italy; ^6^Radiotherapy Unit, Istituto Scientifico Romagnolo per lo Studio e la Cura dei Tumori (IRST) IRCCS, Meldola, Italy

**Keywords:** target alpha therapy (TAT), prostate-specific membrane antigen (PSMA), xerostomia, theragnostic, protectors, dosimetry

## Abstract

Radioligand therapy is a type of internal radiotherapy combining a short-range radioisotope labeled to a carrier with a high affinity for a specific receptor expressed on tumor cells. Targeted alpha therapy (TAT) combines a high-linear energy transfer (LET) emitter (^225^Ac) with a prostate-specific membrane antigen (PSMA) carrier, specifically binding tumor cells in patients with metastatic castration-resistant prostate cancer. Although the antitumor activity of ^225^Ac-PSMA is well-documented, this treatment is nowadays only used as salvage therapy because the high incidence of xerostomia limits the therapeutic window. Thus, methods to reduce salivary toxicity and models able to describe xerostomia incidence are needed. We recently studied the efficacy of salivary gland protectors administered in combination with ^177^Lu-PSMA therapy. Starting from these data, we performed a predictive dosimetric evaluation of ^225^Ac-PSMA to assess the impact of salivary gland protectors in TAT. ^225^Ac-PSMA predictive dosimetry was performed in 13 patients treated with ^177^Lu-PSMA. Sequential whole-body planar images were acquired 0.5–1, 16–24, 36–48, and 120 h post-injection. ^177^Lu time-activity curves were corrected for ^225^Ac physical decay and assumed in equilibrium for all daughters. The OLINDA/EXM spherical model was used for dose estimation of the parotid and submandibular glands. The dose for each daughter was calculated and summed for the total dose estimation. The biologically effective dose formalism was extended to high-LET emitters. For the total biologically effective dose formalism extended to high-LET emitters, including the contribution of all daughter isotopes, the brachytherapy formalism for a mixture of radionuclides was implemented. Equivalent doses in 2 Gy/fraction (EQD2) were then calculated and compared with the normal tissue complication probability model derived from external beam radiotherapy for grade ≥2 xerostomia induction. Median predictive doses were 0.86 Bd_RBE5_/MBq for parotid glands and 1.05 Bd_RBE5_/MBq for submandibular glands, with a 53% reduction compared with previously published data. The results show that the radiobiological model implemented is conservative, as it overestimates the complication rate with respect to the clinical data. Our data shows the possibility of reducing salivary gland uptake in TAT with the coadministration of organ protectors, but these results should be confirmed for TAT with ^225^Ac-PSMA by carrying out prospective trials with defined toxicity endpoints and dosimetry procedures.

## Introduction

The combination of a short-range radioisotope labeled to a carrier/ligand highly specific for receptors expressed on tumor cells enables “internal” radioligand therapy (RLT) to be delivered to tumors. The increased tumor cells turnover and receptor overexpression induces a high isotope concentration within the tumor (1). Internal radiotherapy is also known as radiometabolic treatment (RMT) when iodine-131 is used in thyroid cancer or peptide receptor radionuclide therapy when radiolabeled peptides such as somatostatin analog are used in neuroendocrine tumors (2). Similarly, prostate-specific membrane antigen (PSMA) is an attractive target for diagnosis and therapy of metastasized prostate cancer (3). The PSMA expression is directly correlated with androgen independence, metastasis, and progression. PSMA, also known as glutamate carboxypeptidase II, is a membrane protease anchored in the cell membrane of prostate cancer cells but not in normal prostate cells. A radiolabeled version of a PSMA ligand (Dota-PSMA-617) has been synthesized and has shown promising properties when labeled with ^177^Lutetium, a short-range beta-gamma emitter (4, 5).

PSMA-based RLT is thus becoming an attractive therapeutic option for the clinical management of metastatic castration-resistant prostate cancer patients (3, 4, 6, 7). However, as many as 40% of treated patients do not respond to this β-particle therapy (8). The use of high-linear energy transfer (LET) α-emitters to increase local damage to tumor cells and thus enhance treatment efficacy has aroused widespread interest in this setting. In particular, targeted alpha therapy (TAT) combining ^225^Ac α-emitter with PSMA carrier has proven a promising therapeutic option in terms of disease control for tumors refractory to beta-radiation therapy. Although the antitumor activity of ^225^Ac-PSMA is well-documented (9), this therapy is now only used as salvage therapy because the high rate of irreversible xerostomia limits the therapeutic window. Kratochwil et al. (8, 9) reported their experience in 40 patients treated with ^225^Ac-PSMA administrated with an activity ranging from 50 to 200 kBq/kg. Xerostomia was regularly reported with 100 kBq/kg or more per cycle and was considered intolerable with more than 150 kBq/kg (8). The first symptoms of xerostomia appeared 2–5 days post-TAT, lasting for about 2 months. Partial recovery was observed if no additional cycles were added, but some patients had a chronic loss of secretion function (8).

In a prospective study carried out at Istituto Scientifico Romagnolo per lo Studio e la Cura dei Tumori (IRST) (10), ^177^Lu-PSMA was administered in combination with polyglutamate tablets and ice packs application used as protectors for salivary glands. The gamma emission of ^177^Lu (208 keV, 11% relative abundance) enabled us to perform dosimetry, acquiring serial post-injection whole-body scans. Polyglutamate tablets were orally administered as a substrate for PSMA receptors, and external ice packs were applied to the neck region (3). The dosimetry evaluation performed on 13 patients showed a lower absorbed dose in both parotid and submandibular glands compared with previously published data (11, 12). The efficacy results of the protectors proposed in our study, especially for salivary glands, were encouraging in the context of TAT, as they could potentially improve treatment management, enabling wider use of this therapeutic approach. As the metabolic uptake of a radioisotope is mainly guided by the carrier (*i.e.*, Dota-PSMA-617), it is reasonable to assume that the organ protectors used for ^177^Lu-PSMA could also result in a similar reduction in a predicted dose for ^225^Ac-PSMA treatment. Given that dosimetric imaging is not feasible with ^225^Ac, where lower gamma emission largely impairs post-injection image acquisition, predictive dosimetry was performed assuming an uptake and retention similar to ^177^Lu-PSMA dosimetry. In this way, absorbed dose results for ^177^Lu were converted to ^225^Ac, including the contribution of the decay chain. A radiobiological evaluation was then performed by comparing biologically effective dose (BED) of TAT [relative biological effectiveness (RBE)-weighted] with external beam radiotherapy (EBRT) schedules. The BED formalism was extended to α-emitters (BED_H_) for therapies with a continuous and exponentially decreasing dose rate. To include in the total BED_H_ the contribution of all daughter isotopes in the ^225^Ac chain, the formalism adopted in brachytherapy to estimate the BED for a mixture of radionuclides was implemented. The normal tissue complication probability (NTCP) model derived from EBRT data was then applied to TAT data to estimate the impact of salivary gland protectors on the incidence of acute xerostomia as a function of injected activity.

## Materials and Methods

### Main Treatment and Patient's Population Characteristics

From April 2017 to February 2019, we enrolled 43 patients in the first European phase II RLT prospective trial [EudraCt/RSO number: 2016-002732-32, NCT03454750 (10)] ongoing at our institute (IRST). Patients were stratified on the basis of risk factors. Patients <75 years old unfit to undergo treatment with docetaxel received 5.5 GBq of ^177^Lu-PSMA-617, whereas those who had already been treated with docetaxel, were older than 75 years, or those who had other risk factors received lower activities ranging from 3.7 to 4.4 GBq of ^177^Lu-PSMA-617. Patients received four treatment cycles, with a time interval of 8–12 weeks between 2 consecutive cycles. An additional two cycles were performed for patients with no registered adverse effects, no evidence of progressive disease, and who, in the opinion of the investigator, could obtain a clinical benefit. All patients underwent a pretreatment ^68^Ga-PSMA-HBED-11 whole-body positron emission tomography PET/CT scan. ^177^Lu-Dota-PSMA-617 radiopharmaceutical infusion was performed slowly intravenously in 15–30 min in a dedicated room using a dedicated pump system (patent US 7,842,023 B2). Additional information are provided in Appendix.

Organ-specific drug protectors were administered to reduce organ-at-risk uptake (10–12). For the salivary glands, 30 min before, during, and 4 h after ^177^Lu-PSMA infusion, ice packs were applied to the neck region (3, 13), and patients were given polyglutamate folate tablets of plant origin (Morgan Pharma Monteviale, Italy). To preserve kidney functionality, a 10% mannitol solution in 500 ml was infused before and after ^177^Lu-PSMA injection, 250 ml 30 min before therapy and 250 ml 1 h after therapy (14). Additional organ protectors consisted of eye drops (Naaxia Eye Drop Solution 19.6 mg/0.4 ml, Laboratoires Thea, Clermont-Ferrand, France) to limit lacrimal gland uptake, given 30 min before injection, and laxatives (Movicol, Norgine, Norgine Italia., Milano, Italy) to reduce delayed intestinal uptake, given 24 h post-injection.

### Dosimetry

#### ^177^Lu-PSMA (Prostate-Specific Membrane Antigen) Dosimetry

Serial scintigraphic planar images were acquired 0.5–1, 16–24, 36–48, and 120 h post-infusion. Anterior and posterior images were acquired with a single positron emission computed tomography (SPECT) scanner (Discovery NM/CT 670, General Electric Medical System, Haifa, Israel) equipped with a 3/8″-thick NaI (Tl) crystal with a scan speed of 7 cm/min. The emission energy window was centered on 208 keV (20% width), and additional low and scatter energy windows were centered on 175 and 238 keV (10% width), respectively, and used for scatter correction image. Given that Kratochwil et al. (8) did not perform any attenuation correction to their patents' data, no attenuation correction was applied in this study. Details on the dosimetry protocol were published in previous works (11, 12). Structures of interest for dosimetry evaluation were kidneys, liver, parotid glands (PGs), submandibular glands (SGs), red marrow (RM), and whole body (WB). All structures were delineated on subsequent post-injection planar images, whereas RM dosimetry was based on blood samples. The conjugate projection method (15) was used to evaluate the relative uptake of each considered structure at different time points. For each organ, the time–activity curve was derived for residency time evaluation, and the dose calculation was performed according to the medical internal radiation dose (MIRD) formalism (15, 16) using OLINDA/EXM software (v1.1, Nashville, TN, USA) (17). The OLINDA/EXM adult male phantom was used for WB, kidney, liver, and RM dose estimation. For PGs and SGs, the sphere model of unite density was used. The mass of every single structure was derived on the basis of the pretreatment WB CT scan (^68^Ga-PSMA PET/CT). For paired organs, a mean value between the left and right structures was calculated. The salivary gland dose value was calculated as the mean between PG and SG dose values for each patient. More details are reported in Sarnelli et al. (11). Although the focus of the present study is salivary glands, for the sake of completeness, we reported the predicted dose also for the other considered organs.

#### ^225^Ac-PSMA (Prostate-Specific Membrane Antigen) Predictive Dosimetry

The dosimetric data obtained for ^177^Lu-PSMA evaluation were converted into ^225^Ac-PSMA predictive dosimetry, assuming a similar uptake governed by the PSMA carrier. We used the same method previously published by Kratochwil et al. (8, 18). The ^177^Lu-PSMA time–activity curves were corrected for ^177^Lu physical half-life, and the biological time–activity curves were then used for predictive dosimetry of ^225^Ac. Assuming equilibrium in the decay chain and no translocation during the decay between succeeding disintegrations, the same residence time estimated for ^225^Ac was used for all the daughters in the ^225^Ac chain. S-values specific for each daughter were considered to account for different dose contribution. According to the literature data, an RBE factor equal to 5 was used to weight the α-particle dose contribution concerning the γ and β emission (19). The contributions of α, β, and γ radiations were then summed up for each radioisotope, taking into account the branching ratio of 2% for ^209^Tl and 98% for ^213^Po. As suggested by the MIRD committee, when a deterministic endpoint is considered (19, 20), we expressed data in Barendsen units (Bd) or Bd/MBq. A suffix indicating the RBE value assumed for α-particle weight was added (e.g., Bd_RBE5_ indicates the use of RBE = 5).

The median values of our data were then compared with previously published findings (8, 9). Unfortunately, there is no consensus regarding the choice of unit expression of the RBE-weighted dose. Consequently, it may thus happen that data reported by different studies are derived with the same approach but are expressed with different units (8, 20). For the sake of simplicity, when comparing our data with those from other studies, we used the unit of Sv_RBE5_/MBq according to (8).

### Radiobiological Model

#### BED (Biological Effective Dose) Calculation

When comparing the effect of delivered dose with high- or low-LET radiation, the different ability to create biological damage per unit of delivered dose should be taken into account. The radiosensitivity parameters used in the linear-quadratic (LQ) model for high-LET radiation are therefore different from those used for low-LET. The concept of maximum RBE (RBE_M_) is used to incorporate this effect for the linear component of the LQ model in the BED calculation, maximizing the RBE value on cell survival curves (21). We derived RBE_M_ from the Equation (8) in (22) as follows:

(1) $$\begin{array}{r} RBE_{M}=RBE_{exp}+\frac{d}{\alpha /\beta }\left(\frac{RBE_{exp}^{2}-1}{RBE_{exp}}\right) \end{array}$$

where RBE_exp_ is the experimental value of high-LET radiation assumed from literature (19), d is the fractional dose of the reference low-LET radiation, α/β is the ratio of the dose-rate-independent and the dose-rate-dependent term in the LQ model estimated for low-LET radiation (23).

Aiming to compare the effect of TAT delivered dose with the low-LET EBRT schedules, the BED (24, 25) for high-LET (BED_H_), continuous and exponentially decreasing dose rate, was calculated using the equation proposed by Dale and Jones (21). The formalism derived for brachytherapy implants with a mixture of radionuclide was used (26–29) to account for all daughter isotopes in the ^225^Ac decay chain:

(2) $$\begin{array}{r} BED_{H}=\frac{1}{\lambda }\sum_{n}{\left(R_{0}\right)}_{n}\left\{RBE_{M}+\frac{\sum_{n}\sum_{p}{\left(R_{0}\right)}_{n}{\left(R_{0}\right)}_{p}}{\left(\lambda +\mu \right)\left(\alpha /\beta \right)\left[\sum_{n}{\left(R_{0}\right)}_{n}\right]}\right\} \end{array}$$

were n and p denote the different daughter in the decay chain, λ the effective half-life (a combination of ^225^Ac physical and biological half-lives, assumed equal for all the daughter isotopes), and μ the repair time. (R_0_)_n_ is the initial dose rate expressed as follows:

(3) $$\begin{array}{r} {\left(R_{0}\right)}_{n}\left[\frac{mGy}{h}\right]=D_{n}\left[\frac{mGy}{MBq}\right]A_{i}\left[MBq\right]\;\lambda \left[\frac{1}{h}\right] \end{array}$$

where D_n_ is the non-RBE-weighted predicted absorbed dose and A_i_ the injected activity.

For bi-exponential curve fitting, the λ value corresponding to the slow washout phase (lower value) was used for BED_H_ calculation. By introducing RBE_M_, the calculated BED_H_ remains compatible with the LQ model and is expressed in the same biological units as for low-LET calculation (Gy), allowing a direct comparison with EBRT schedules (22). For the BED_H_ calculation, we considered only the α emissions, whereas the β and γ emissions were neglected.

We compared our data with those derived from EBRT schedules for both late [at 1-year post-EBRT, QUANTEC data (30, 31)] and early [at 3-month post-EBRT, Strigari et al. (32)] xerostomia post-treatment. For this purpose, we calculated the BED for low-LET radiation (BED_L_) of EBRT as (33):

(4) $$\begin{array}{r} BED_{L}=D_{L}\left(1+\frac{D_{L}/N}{\alpha /\beta }\right) \end{array}$$

where D_L_ is the total dose for low-LET EBRT schedules, and N is the number of fractions.

As QUANTEC (30, 31) reports as dose constraint for xerostomia induction a value calculated on PGs alone, we did not include in this comparison the SG dose values. Whereas, when comparing our data to the model of Strigari et al. (32) that includes both PGs and SGs, the mean value between them was considered for salivary glands dose.

#### NTCP (Normal-Tissue Complication Probability) Modeling

To compare the data with the NTCP model, the equivalent dose in 2 Gy/fr (EQD2) is calculated as (34):

(5) $$\begin{array}{r} EQD2=\frac{BED_{H}}{\left(1+\frac{2\;Gy}{\alpha /\beta }\right)} \end{array}$$

The Lyman–Kutcher–Burman formalism (35, 36) was used for the NTCP model as:

(6) $$\begin{array}{r} NTCP=\frac{1}{\sqrt{2\pi }}\int_{0}^{t}e^{-x^{2}/2}dx \end{array}$$

(7) $$\begin{array}{r} t=\frac{EQD2-TD50}{m^{*}TD50} \end{array}$$

where TD50 is the tolerance dose for a homogenous dose distribution to the organ in which 50% of the patients are likely to experience severe xerostomia, and m is the slope of the dose–response curve.

#### Data Analysis

In accordance with the clinical protocol active in our institute, all enrolled patients received salivary gland protectors in combination with ^177^Lu-PSMA. For this reason, no data without drug protectors were available in our patient cohort. To compare the results of predictive dosimetry for ^225^Ac-PSMA obtained in our patient cohort with those obtained without salivary gland protectors (8), the predictive dose calculated for our patients was rescaled according to the ratio between the mean predicted dose value for salivary glands reported in the study by Kratochwil et al. (8) and the same value estimated in our patient cohort. The ${\text{BED}}_{\text{H}}^{\prime }$ was then calculated for rescaled predictive doses as previously described. The EBRT-derived NTCP model was then used to estimate the probability of xerostomia for ^225^Ac-PSMA with and without the administration of the salivary gland protector. Moreover, based on BED_H_ dependence on injection activity, the impact of different activity concentration levels was evaluated, scaling from 50 to 200 kBq/kg.

## Results

Predictive dosimetry evaluation was performed on 13 patients enrolled in the ^177^Lu-PSMA protocol (nine at the first cycle and four at the second cycle).

### Predictive Dose for ^225^Ac-PSMA

Median (range) mass values of considered structures were 371 g (223–628) for kidneys, 1,830 g (1,132–2,366) for liver, 53 g (33–89) for PGs, 17 g (13–34) for SGs, and 80 kg (72–105) for WB. For paired organs, the sum of the left and right organ is reported. Median (range) effective half-lives were 17.6 h^−1^ (0.07–46.2) for kidneys, 12.1 h^−1^ (4.2–33.8) for liver, 25.5 h^−1^ (1.7–46.2) for PGs, 10.0 h^−1^ (2.8–32.7) for SGs, 2.1 h^−1^ (0.5–15.2) for RM, and 5.7 h^−1^ (1.4–17.0) for WB. The mean contribution (range) values to the total predictive dose of each single particle emission were 99.47% (98.20–99.79), 0.49% (0.22–1.76), and 0.05% (0.02–0.15) for α, β, and γ, respectively. Median (range) predictive doses were 0.67 Bd_RBE5_/MBq (0.15–1.81) for kidneys, 0.11 Bd_RBE5_/MBq (0.02–0.24) for liver, 0.86 Bd_RBE5_/MBq (0.49–2.43) for PGs, 1.05 Bd_RBE5_/MBq (0.42–1.98) for SGs, 0.07 Bd_RBE5_/MBq (0.03–0.14) for RM, and 0.04 Bd_RBE5_/MBq (0.02–0.11) for WB (Table 1).

**Table 1:** Results of ^225^Ac-PSMA predictive dosimetric study in terms of Bd_RBE5_/MBq (11).

	**Kidneys** ** (Bd_**RBE5**_/MBq)**	**Liver** ** (Bd_**RBE5**_/MBq)**	**Parotid glands** ** (Bd_**RBE5**_/MBq)**	**Submandibular glands** ** (Bd_**RBE5**_/MBq)**	**Red marrow** ** (Bd_**RBE5**_/MBq)**	**Whole body** ** (Bd_**RBE5**_/MBq)**
Patient 1	0.67	0.23	1.28	1.52	–	0.11
Patient 2	0.54	0.17	2.43	1.98	0.08	0.05
Patient 3	1.81	0.08	0.64	0.79	0.08	0.04
Patient 4	0.15	0.02	0.70	1.05	0.03	0.02
Patient 5	0.86	0.23	1.16	0.87	0.08	0.04
Patient 6	0.42	0.06	0.86	1.09	0.14	0.09
Patient 7	0.57	0.12	0.57	1.15	0.07	0.02
Patient 8	0.70	0.14	0.52	0.96	0.03	0.03
Patient 9	0.45	0.09	0.49	1.30	0.05	0.04
Patient 10	0.54	0.08	1.11	0.83	0.05	0.04
Patient 11	0.67	0.09	1.39	0.68	–	0.03
Patient 12	1.06	0.24	1.83	1.89	–	0.09
Patient 13	0.73	0.11	0.50	0.42	–	0.02
Median (range)	0.67 (0.15–1.81)	0.11 (0.02–0.24)	0.86 (0.49–2.43)	1.05 (0.42–1.98)	0.07 (0.03–0.14)	0.04 (0.02–0.11)
Mean (SD)	0.71 (0.40)	0.13 (0.07)	1.04 (0.59)	1.12 (0.46)	0.07 (0.03)	0.05 (0.03)

*Data were extrapolated from ^177^Lu-PSMA dosimetry evaluations. OLINDA/EXM adult male phantom was used for the whole-body, kidney, liver, and red marrow dose estimation, whereas spherical model was used for parotid and submandibular glands. Blood sample data were not available for patients 1, 11, 12, and 13. SD, standard deviation*.

**Figure 1:**
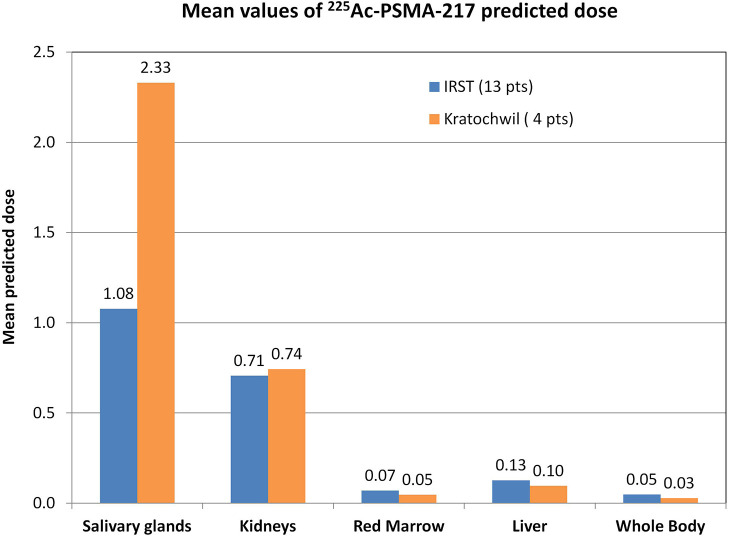
Graphical comparison of our data (13 patients) with data published by Kratochwil et al. (8) (4 patients). Mean values of the predicted dose calculated for each patient group. Data were extracted from whole-body planar images and blood sample acquisition and analyzed in the same way. Our data are reported in Bd_RBE5_/MBq, as suggested by the MIRD committee for deterministic effects (20), whereas data of Kratochwil et al.'s study (8) are reported in Sv_RBE5_/MBq in accordance with their published paper.

Figure 1 compares our data with those of Kratochwil et al.'s study (8). The reduced absorbed dose observed with ^177^Lu-PSMA dosimetry (11, 12) was also confirmed for ^225^Ac-PSMA predictive dosimetry, with a 53% decreased of predicted dose in salivary glands in our patient group [1.08 vs. 2.33 Sv_RBE5_/MBq (8), mean values].

### Comparison With EBRT Biological Model

Table 2 reports the values of parameters used for BED calculation. Considering xerostomia as a toxicity endpoint, a value of α/β equal to 3 Gy was used (37, 38), whereas a value of 0.46 h^−1^ was used for the μ (39, 40). For high-LET radiation, assuming RBE_exp_ = 5 (19), the RBE_M_ was 8.2. Fractional dose d of the reference low-LET radiation was assumed to be 2 Gy. As predictive dosimetry, an injection activity of 100 kBq/kg was assumed on the basis of single patient weight (8).

**Table 2:** Value of parameters used for BED evaluation.

**Parameter**	**Value**	**Note (reference)**
α/β	3 Gy	Xerostomia (37, 38)
μ	0.46 h^−1^	Repair time (39, 40)
^225^Ac-PSMA injected activity	100 kBq/kg	(8)
RBE_exp_ high-LET radiation	5	(19)

Considering this RBE_M_ value and an injection activity of 100 kBq/kg, the BED_H_ was calculated for the predictive dose values reported in *Predictive Dose for* ^225^*Ac-PSMA With a Prostate-Specific Membrane Antigen*. Median (range) BED_H_ values were 36.5 Gy (12.4–237.0) for PGs, 55.0 Gy (12.1–203.9) for SGs, and 51.9 Gy (15.9–220.4) for both salivary glands (Table 3). A QUANTEC dose constraint of D_L_ = 26 Gy on PGs delivered in N = 30 fractions was considered for EBRT (30, 31) for late xerostomia induction at 1-year post-EBRT. This dose constraint corresponds to a BED_L_ of 33.5 Gy. For 100 kBq/kg injection activity, 7 of our 13 patients, the PG BED_H_ was >33.5 Gy. Rescaling data to Kratochwil et al.'s (8) mean predicted dose value (*i.e.*, corresponding to a patient population without salivary gland protectors), the ${\text{BED}}_{\text{H}}^{\prime }$ for all 13 patients was >33.5 Gy (data not shown).

**Table 3:** BED_H_ calculated for parotid and submandibular glands.

	**Parotid** ** glands** ** (Gy)**	**Submandibular glands** ** (Gy)**	**Salivary** ** glands** ** (Gy)**
Patient 1	56.6	57.8	57.2
Patient 2	237.0	203.9	220.4
Patient 3	15.4	22.0	18.7
Patient 4	42.1	140.5	91.3
Patient 5	36.5	15.7	26.1
Patient 6	22.9	33.8	28.3
Patient 7	27.6	104.7	66.2
Patient 8	22.2	55.0	38.6
Patient 9	12.4	91.3	51.9
Patient 10	49.0	23.4	36.2
Patient 11	125.9	34.0	80.0
Patient 12	142.2	138.2	140.2
Patient 13	19.7	12.1	15.9
Median (range)	36.5 (12.4–237.0)	55.0 (12.1–203.9)	51.9 (15.9–220.4)
Mean (SD)	62.3 (66.5)	71.7 (59.8)	67.0 (57.6)

*Salivary gland BED_H_ is calculated as mean of parotid and submandibular gland BED_H_ values. Used parameters are reported in Table 2. SD, standard deviation*.

**Figure 2:**
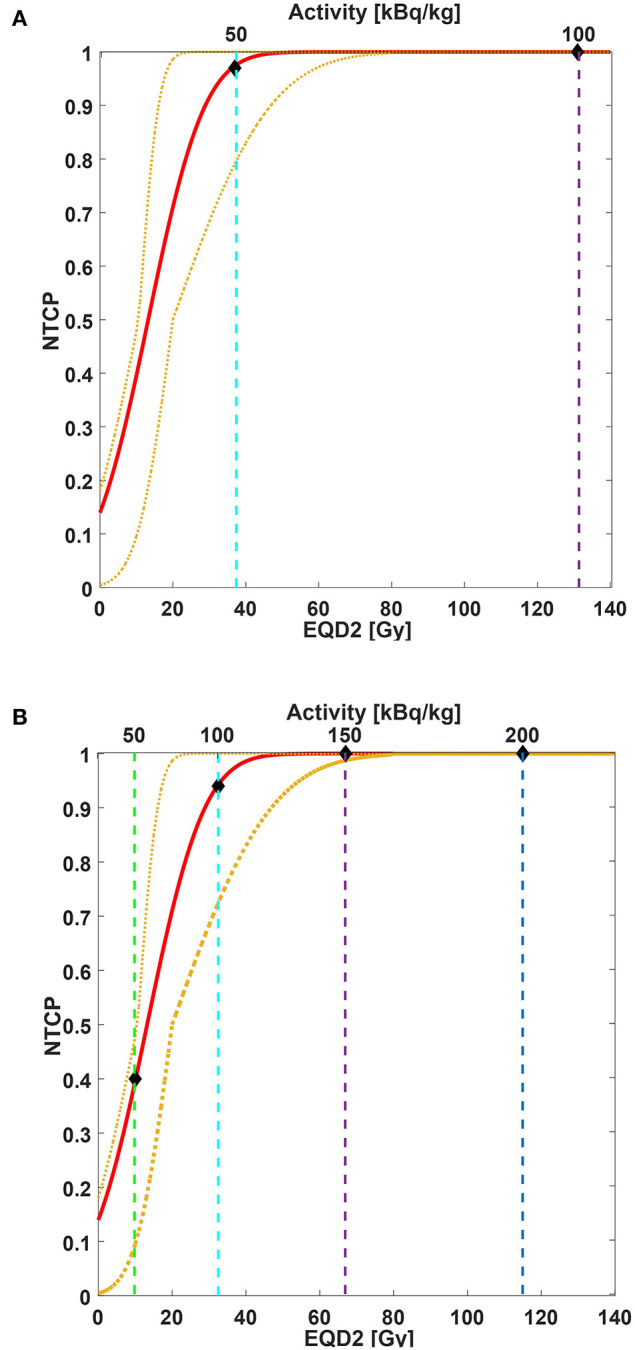
NTCP curve (red line) of acute xerostomia incidence as a function of mean EQD2 values (bottom x-axis) and injected activity (top x-axis). Yellow dotted lines are 95% CI. NTCP model parameters: TD%50 = 14 Gy_EQD2_, m = 0.88. Considered endpoint: grade ≥ 2 xerostomia 3-month post-EBRT. Considered structures: salivary glands (parotid and submandibular glands together). Both EBRT and TAT doses were converted into EQD2. For EQD2 calculation: **(A)** predictive dose value estimated for our patient cohort and rescaled to Kratochwil et al.'s (8) mean predicted dose value is used; **(B)** predictive dose estimated for our patient cohort is used. Vertical dashed lines correspond to 50 kBq/kg (green line), 100 kBq/kg (cyan line), 150 kBq/kg (purple line), and 200 kBq/kg (blue line). Diamonds indicate the estimated incidence of acute xerostomia based on EBRT NTCP modeling for the injected activities of interest.

We used Strigari et al.'s data for NTCP modeling (32). In this model, the dose to the salivary glands is the mean dose of both PGs and SGs and converted into EQD2. Considering a salivary flow reduction of <45% of the initial value at 3-month post-EBRT (grade ≥ 2, G2+) as an endpoint, the fitting parameters were TD50 = 14 Gy_EQD2_ and m = 0.88 [personal communication (32)]. Figure 2 shows the comparison between the considered NTCP model and the data rescaled to Kratochwil et al.'s (8) mean predicted dose value (Figure 2A) and our data (Figure 2B). The different activity concentration levels are also indicated. Without the administration of salivary gland protectors, the predicted probability values of acute G2+ xerostomia based on the NTCP model were 97% (95% CI: 79–100%) for A_i_ equal to 50 kBq/kg and 100% (95% CI: 99–100%) for A_i_ equal to 100 g, 150, and 200 kBq/kg (Figure 2A). The predicted incidence values of xerostomia for TAT combined with salivary gland protectors were 40% (95% CI: 10–48%) for A_i_ equal to 50 kBq/kg, 94% (95% CI: 72–100%) for A_i_ equal to 100 kBq/kg, 100% (95% CI: 99–100%) for A_i_ equal to 150 kBq/kg, and 100% (95% CI: 100–100%) for A_i_ equal to 200 kBq/kg (Figure 2B).

## Discussion

The predictive dosimetry of ^225^Ac-PSMA confirms the reduction of absorbed dose previously reported in our protocol with ^177^Lu-PSMA in combination with folic glutamate tablets and ice pack application as salivary gland protectors (11, 12). The conversion from a β/γ emission to an α/β/γ emission could not be calculated with a global scaling factor between ^177^Lu and ^225^Ac emissions. In fact, the conversion of ^177^Lu emission into the decay chain of ^225^Ac includes daughters emitting α, β, and γ radiations with different branching ratios and different RBE values. Therefore, the scaling factor takes into account the emission of each daughter and is a linear combination of the different branching ratios of the radiations emitted weighted by the corresponding RBE factor [see Supplementary Table 4 of Supplementary Material in reference (18)]. We obtained a reduction of 53% in predicted dose compared with previously published data, i.e., 1.08 vs. 2.33 Sv_RBE5_/MBq (8). Kratochwil et al. (8, 9) did not report any use of salivary gland protectors in their study. Therefore, our results differ from those of Kratochwil et al.'s study (8) for the potential sparing effect of both ice pack application and polyglutamate folate administration. The data from both studies are derived with the same dosimetry protocol and procedure and are therefore comparable. Acute xerostomia was identified by Kratochwil et al. (8, 9) as major toxicity impairing treatment, and the authors experimentally identified 100 kBq/kg as an activity concentration threshold capable of avoiding acute toxicity. Kratochwil et al. (8) reported the xerostomia incidence stratified with injection activity only in a subgroup of 16 patients. In particular, four patients were injected with 50 kBq/kg, and none experienced severe xerostomia; four patients were injected with 100 kBq/kg, and none experienced severe xerostomia; two patients were injected with 150 kBq/kg, and one experienced severe xerostomia; and four patients were injected with 200 kBq/kg, and three experienced severe xerostomia.

Comparing our data with the acute tolerance threshold of 17 Sv_RBE5_ reported in Kratochwil et al. (8, 18), we did not expect to find a high-grade xerostomia incidence in our treatment for 100 kBq/kg ^225^Ac-PSMA injected activity. At the same time, when the EBRT model of acute xerostomia was applied to our data rescaled to Kratochwil's (8) mean predicted dose value, the expected incidence rate was higher than that observed by Kratochwil's group (8). The large discrepancy observed between the clinical data of Kratochwil's study (8) and our model derived from the combination of EBRT and brachytherapy formalisms may arise from different factors.

First of all, we should consider the limited cohort of patients administered ^225^Ac-PSMA for whom toxicity data were evaluated and stratified with injected activity (16 patients), resulting in a fairly large error in the observed toxicity incidence (8). In addition, we scaled our data with a factor equal to the ratio between the mean value of our population and the one reported in Kratochwil et al.'s (8) study, calculated on dosimetric data for four patients. However, the reduced number of patients included in the dosimetry study of Kratochwil's group (8) (4 patients) may strongly impact the calculated scaling factor between the two patients' populations. Therefore, NTCP values calculated on the rescaled patient population should be considered with caution. In addition, the attenuation correction, which was not considered in the dosimetric analysis, may play a relevant role to account for the discrepancy between the predicted and observed toxicity.

Despite the above issues, other radiobiological considerations may affect the biological evaluation of toxicity induction based on predictive dosimetry estimations for TAT therapy from ^177^Lu-PSMA data. We assumed that there would be a similar local uptake and temporal distribution between ^177^Lu-PSMA and all daughters of the decay chain of ^225^Ac-PSMA, mainly governed by the PSMA carrier. Although this assumption may be considered sufficiently robust if ^177^Lu-PSMA and ^225^Ac-PSMA are considered alone, it is no longer valid for all daughters in the decay chain. In fact, if the link between ^225^Ac daughters and the PSMA carrier is no longer stable, a significant redistribution of daughter nuclides may occur throughout the body, and, consequently, their uptake may substantially differ from ^177^Lu-PSMA distribution (41).

Moreover, although a uniform dose distribution was assumed inside each salivary gland, the pre-therapy ^68^Ga-PSMA-HBED-11 PET/CT (Supplementary Figure S1) clearly showed that this was not the case. The standard uptake value distribution of ^68^Ga-PSMA radiotracer could be considered a surrogate of ^225^AC-PSMA uptake. Taking into account that the dose delivered in TAT could be considered extremely localized, the non-uniform ^68^Ga-PSMA standard uptake value distribution could, therefore, also be considered a surrogate of TAT dose distribution. Supplementary Figure S3 depicts the dose distribution and Supplementary Figure S4 the dose–volume histogram (DVH) of PGs and SGs in a head-and-neck cancer patient who underwent EBRT. Thanks to the new technology of intensity-modulated radiotherapy (IMRT), EBRT allows sparing a portion of salivary glands and results in a non-uniform dose distribution inside PGs. Beyond the non-uniform distribution, one noticeable aspect is that the portions of salivary glands receiving the high doses in the case of ^68^Ga-PSMA (Supplementary Figures S1, S2) are different from the high-dose region in EBRT (Supplementary Figures S3, S4). For this reason, the considerations achieved on toxicity impact based on tissue damage assumed from EBRT dose distribution should be carefully managed when applied to RLT. A three-dimensional (3D) approach would be favorable to take into account these differences. However, the dosimetry approach based on 2D planar images does not permit differences in regional uptake to be seen, and the dose evaluation is therefore limited to the mean predicted dose evaluation. It is also well-known from EBRT experiences that both the mean dose to PGs and the DVH constraints should be taken into account to reduce the impact of xerostomia caused by the volume effect for parallel organs (42–44). None of these parameters can be evaluated without 3D information on activity uptake. A 3D SPECT imaging with ^177^Lu-PSMA centered on the neck region is needed to be able to calculate dose distribution and generate a DVH. The uptake information for each voxel derived from 3D SPECT ^177^Lu imaging can then be converted into ^225^Ac emission with the same formalism mentioned earlier. However, the limited field-of-view of traditional SPECT scanners does not allow for 3D imaging acquisition of the WB, and SPECT acquisition is generally only centered in the abdominal region to evaluate the dose to the kidneys, other important dose-limiting organs in RLT (45). Fortunately, new SPECT scanners are beginning to emerge that are capable of providing full 3D SPECT imaging along the WB by combining a 3D acquisition with a dynamic longitudinal motion of the patient couch. Another solution could be to perform a hybrid dosimetry evaluation where time–activity curves are evaluated on serial planar images, and the 3D dose distribution is evaluated in a single 3D SPECT acquired at a single time-point post-injection (45). Despite this, even with the conversion of ^177^Lu 3D dose distribution into ^225^Ac, another factor to take into consideration is that the local damage performed by low-LET β-particle and γ-emitters is different from the one of α-particle, characterized by clusters of spots with high-energy deposition (8, 46). The presence of hot spots may dramatically change the pattern of tissue damage when changing from a low-LET β/γ-emitter to a high-LET α-emitter, and this difference should also be taken into account.

With regard to the salivary gland composition, it is known that they are mainly composed of adipose tissue, ductal, and acinar cells, able to produce saliva. Histopathological studies of patients treated with EBRT to the neck region have shown that irradiation of salivary glands results in a loss of the acinar cell component (47), which correlates with both volume reduction of the glands and decreased saliva flow. Moreover, van Luijk et al. (48) found that the recovery of radiation-induced xerostomia can be repaired by a pool of stem cells, mainly located in the central region of PGs. The authors showed that the protection of this central zone during EBRT in head-and-neck patients enables organ function to be preserved. All of these factors may play an important role in the functional damage induced by tissue irradiation and warrant further investigation. We considered salivary glands uniform in their composition and radiosensitivity. To better understand the underlying radiobiological process of damage, the interaction between short-range high-LET α-particle and different salivary gland tissue components should be investigated with microdosimetry and autoradiography (19).

Assumptions were also made about the parameters used in BED and NTCP calculation. In a first approximation, we considered a mono-exponential curve fitting capable of describing the long-term organ washout. However, time–activity curves are generally best fitted with bi-exponential curves. An improved model would consider both λ parameters of bi-exponential curve fitting. Furthermore, in the BED_H_ formalism for the mixture of radionuclide, we assumed the ^225^Ac λ-value valid for all the daughters in the decay chain. In fact, in our case, the mixture is produced as a consequence of the ^225^Ac decay, which has life-time significantly longer than one of all the daughter isotopes. Therefore, we assume that the decay rates of all daughter isotopes are dominated by the one of ^225^Ac.

Moreover, it is important to point out that in our model, the RBE_exp_ was assumed equal to 5, in accordance with previously published studies and MIRD recommendations (19). However, this value, which was extracted from *in vitro* experiments, has never been clinically validated in human subjects (49). For this reason, some authors (49, 50) prefer not to apply an RBE factor to the calculated dose. In our radiobiological model, we adopted the formalism proposed by Dale for BED for high-LET particle, including the RBE_M_ (22). Carabe-Fernandez et al. (51) published a model that was also the minimum value of RBE (RBE_min_) and is included in the BED formalism. They found that the dependence of BED from RBE_min_ has a larger impact on acute toxicity incidence than for the late one. In fact, the RBE_min_ could assume values less than unity, reducing the contribution of the quadratic term in the BED formula. The dependence of BED for a high-LET particle on both RBE_M_ and RBE_min_ may explain the observed discrepancy between model and clinical data in our study. Further improvements of the present model should consider both terms, RBE_M_ and RBE_min._

Furthermore, the RBE experimental values provided in the literature for α-particles are measured with a single emitter. To our knowledge, no RBE experimental data are reported in the literature for a mixture of different radionuclides with different half-life and the emission of α-particles of different energies.

Lastly, it is possible that the high-dose rate involved with ^225^Ac TAT could potentially shift the radiobiology effect in a region of RBE plot of overkilling (52). Prospective clinical studies are therefore required to be able to provide clinical data of toxicity impact in combination with dosimetric data.

Palm et al. (19, 53) found that the polonium can diffuse away from the decay site, reducing, therefore, the local damage. Inside the ^225^Ac decay chain, the ^213^Po contributes to 30% of the absorbed dose in the sphere model. By removing the ^213^Po contribution from the radiobiological calculation of the rescaled patient population to the Kratochwil et al. (8) data, the EQD2 of salivary glands for 100-kBq/kg injected activity is reduced of 66%. The corresponding NTCP values then shifted to 71% (95% CI: 50–99%) for A_i_ equal to 50 kBq/kg, 100% (95% CI: 99–100%) for A_i_ equal to 100 kBq/kg, and 100% (95% CI: 100–100%) for A_i_ equal to 150 and 200 kBq/kg (data not shown). Even with the correction proposed by Palm et al. (53), the model remains conservative, as it overestimates the clinical data of Kratochwil et al.'s study (8). A model able to describe the source–target interaction at the microscopic level is therefore required to improve the agreement between the clinical data and the theoretical model (19, 54). As suggested by Kvinnsland et al. (54), a microdosimetry evaluation that takes into account both the energy spectrum and intracellular differences can describe the underplaying biological process in detail, whereas a mean value may not be sufficiently representative.

Finally, the NTCP model based on EBRT also has different issues that should be carefully taken into account. First, there is no consensus regarding TD50 and m fitting parameters in different studies, with values spanning over a wide range (TD50 = 28.4 to 52 Gy, m = 0.10–0.40 for late xerostomia induction) (55). This is due to substantial variability in study design such as differences in treatment modality and dose distribution, dose reporting of the single spared PG or a mean value over both, inclusion or not of SGs and/or oral cavity, salivary measurement methods, considered endpoint, segmentation, inter-gland sensitivity, and/or patient geographical location (55). Given that both PGs and SGs are irradiated in TAT, we compared our data with those obtained using the model developed by Strigari et al. (32), which has the advantage of including both PG and SG mean dose and considering acute grade ≥ 2 xerostomia at 3-month post-EBRT as an endpoint (personal communication).

Methods for salivary glands protection have previously been implemented for both PSMA-based therapy [^177^Lu-PSMA RLT (3, 56)] and imaging [^68^Ga-PSMA PET/CT (57)]. However, the efficacy of these methodologies in TAT has never been investigated before, and no toxicity modeling was tested with these settings.

The results we obtained on the mean predictive dose reduction when using protectors specific for salivary glands in combination with ^177^Lu-PSMA therapy would also appear promising for TAT. However, our results should be confirmed with ^225^Ac-PSMA therapy data and post-injection evaluation of toxicity and treatment outcome. BED calculation and NTCP modeling overestimate the incidence of high-grade xerostomia reported in some studies, suggesting that further elements should be included in the biological model of tissue damage induced by TAT. Further investigation and appropriate modeling are warranted to describe better the underlying radiobiological process of damage from high-LET therapy. This can be done by carrying out prospective trials with defined toxicity endpoints and dosimetry procedures. At the same time, appropriate NTCP biological models specific for TAT should be developed based on clinical data.

## Data Availability Statement

The datasets generated for this study are available on request to the corresponding author.

## Ethics Statement

The studies involving human participants were reviewed and approved by Ethics Committee of Area Vasta Romagna and by the competent Italian regulatory authorities (Ethical approval no. 1704 of 15.02.2017, Protocol IRST 185.03). The patients/participants provided their written informed consent to participate in this study.

## Author Contributions

MLB: dosimetry, data analysis, and writing. AS: study conception and critical revision of the manuscript for intellectual content. FC: data analysis. EM: dosimetry. GP, FM, and PC: image analysis and diagnosis. VDI: radiopharmaceutical preparation. LS: EBRT modeling. GP, SS, SN, MC, and AR: patient management. All authors: contributed to the article and approved the submitted version.

## Conflict of Interest

The authors declare that the research was conducted in the absence of any commercial or financial relationships that could be construed as a potential conflict of interest.

## Supplementary Material

Supplementary Figure 1 Pre-treatment ^68^Ga-PSMA-HBED-11 PET/CT image. The non-uniform uptake distribution inside parotid glands is clearly visible.

Supplementary Figure 2 Cumulative histogram of standard uptake value (SUV) of parotid and submandibular glands.

Supplementary Figure 3 Dose distribution of a head-and-neck cancer patient treated with helical TomoTherapy (66, 60, and 45 Gy in 30 fractions). Red contour for 60 Gy target, dark-red and cyan contours for right and left PGs respectively. Mean absorbed dose: 25.3, 25.9, 50.6, and 50.1 Gy for right and left PGs, right and left SGs respectively.

Supplementary Figure 4 Dose-volume histogram (DVH) of parotid and submandibular glands.

## Appendix

The study, conducted in accordance with the Declaration of Helsinki and good clinical practice (guidelines, was approved by the Ethics Committee of Area Vasta Romagna and by the competent Italian regulatory authorities (Ethical approval no. 1704 of 15.02.2017, Protocol IRST 185.03). All patients gave written informed consent. Admission criteria were radiological progression (in soft tissue or bone) or biochemical progression (sequence of three prostate-specific antigen rising values from a screening prostate-specific antigen value ≥ 2 ng/ml) according to Prostate Cancer Working Group 3 in the pre-study period, refractory or unfit to conventional standard treatments (hormonal or chemotherapeutic treatment such as abiraterone, enzalutamide, and docetaxel). Other eligible criteria were age ≥18 years; histological or cytological confirmation of advanced castration-resistant prostate cancer [Prostate Cancer Working Group 3 criteria (58)]; measurable disease (RECIST 1.1 criteria); Eastern Cooperative Oncology Group performance status <2 (59); adequate hematological, liver, and renal functions: hemoglobin ≥9 g/dl, absolute neutrophil count ≥1.5 × 10^9^/L, platelets ≥100 × 10^9^/L, bilirubin ≤ 1.5 × upper normal limit (UNL), alanine aminotransferase and aspartate aminotransferase <2.5 × UNL (<5 × UNL in the presence of liver metastases, creatinine <2 mg/dl). Patients treated with chemotherapy and^223^Ra radiotherapy within 4 weeks, treated within 2 weeks with palliative radiotherapy, or with the persistence of acute toxicities from any prior therapy (grade >1, Common Terminology Criteria for Adverse Events version 4.03) were excluded.

National good preparation standards [NBP MN (60)] for pharmaceutical products were followed for^177^Lu-PSMA-617 production, as required by current Italian legislation. Dota-PSMA-617 was kindly provided by Endocyte Inc. (West Lafayette, IN 47906, USA), and^177^Lu was purchased from AAA Severijns (LuMark®, Baarle-Nassau, The Netherlands) or ITG (Endolucinbeta®, Isotope Technologies Garching GmbH, Garching, Germany). The labeling procedure and quality control of the^177^Lu-DOTA-PSMA-617 compound were performed in the Radiopharmacy Laboratory of our Institute (11) (IRST Istituto di Ricovero e Cura a Carattere Scientifico, Meldola, Italy).
