# Supplementary figures and images for: Targeted Alpha Therapy in mCRPC (Metastatic Castration-Resistant Prostate Cancer) Patients: Predictive Dosimetry and Toxicity Modeling of 225Ac-PSMA (Prostate-Specific Membrane Antigen)
Source: Front Oncol. 2020 Nov 5;10:531660. doi: 10.3389/fonc.2020.531660 (PMC7674768; doi:10.3389/fonc.2020.531660)

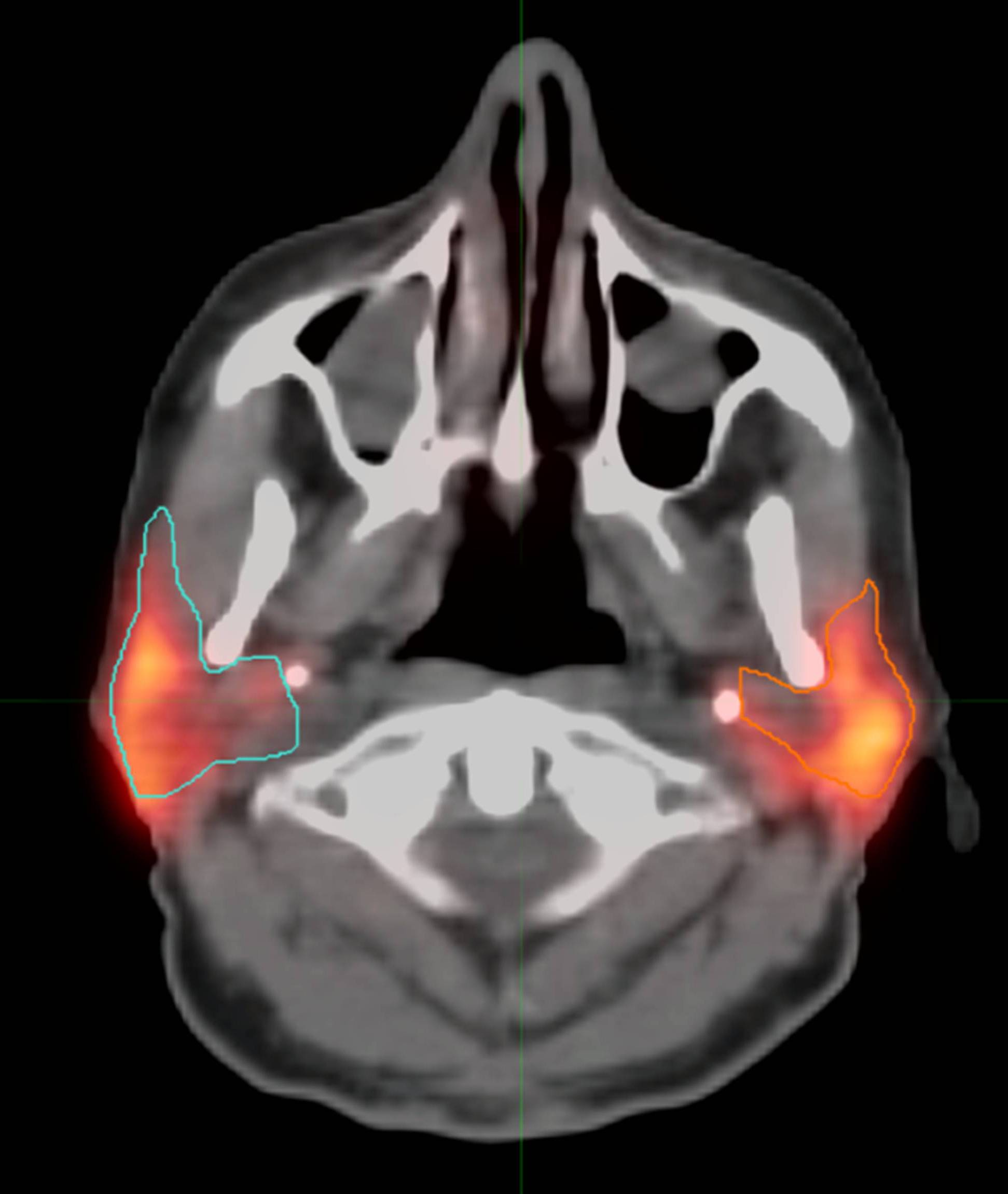

Supplement: Supplementary Figure 1 — Pre-treatment 68Ga-PSMA-HBED-11 PET/CT image. The non-uniform uptake distribution inside parotid glands is clearly visible. [file Image_1.JPEG]

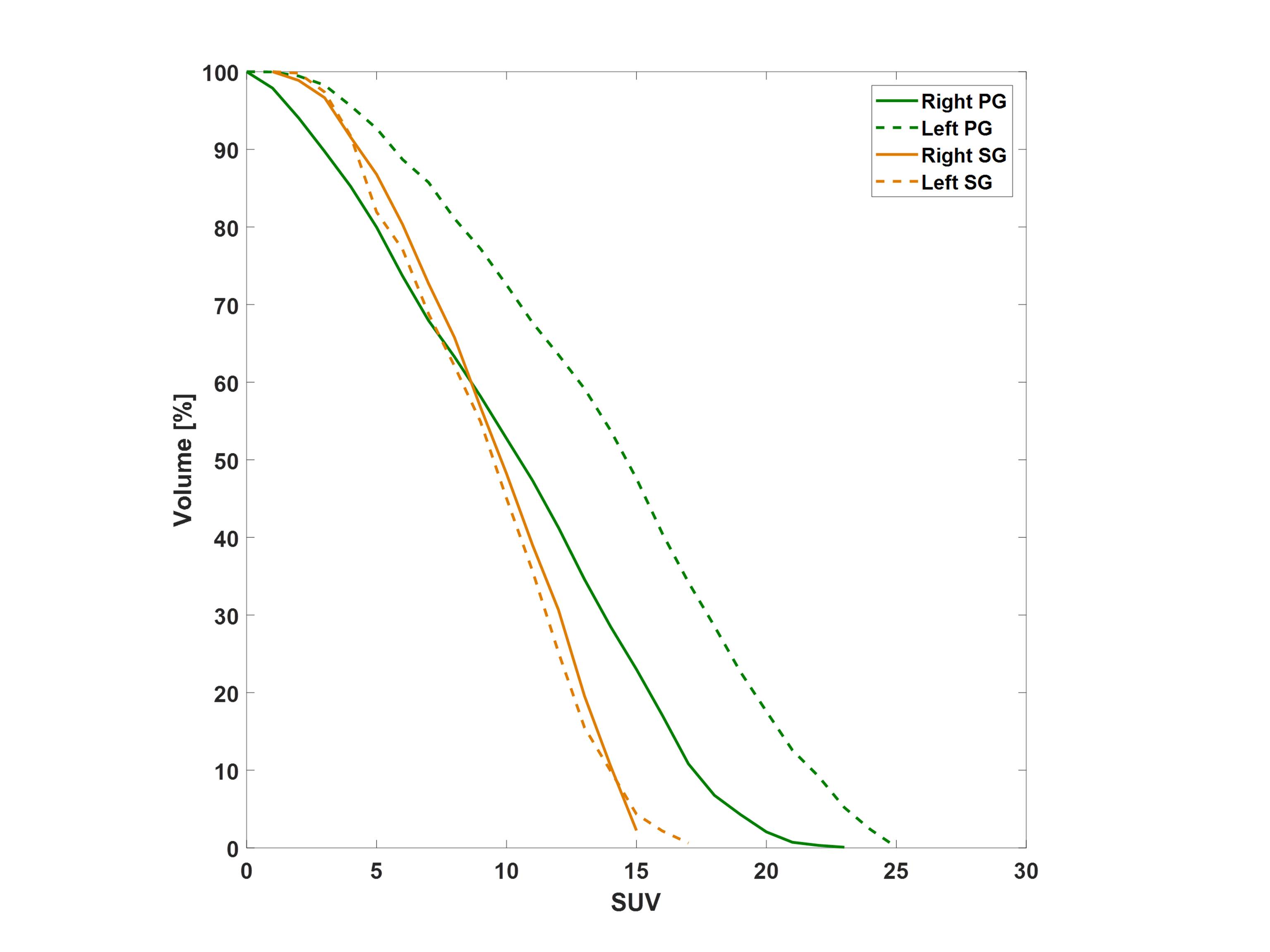

Supplement: Supplementary Figure 2 — Cumulative histogram of standard uptake value (SUV) of parotid and submandibular glands. [file Image_2.JPEG]

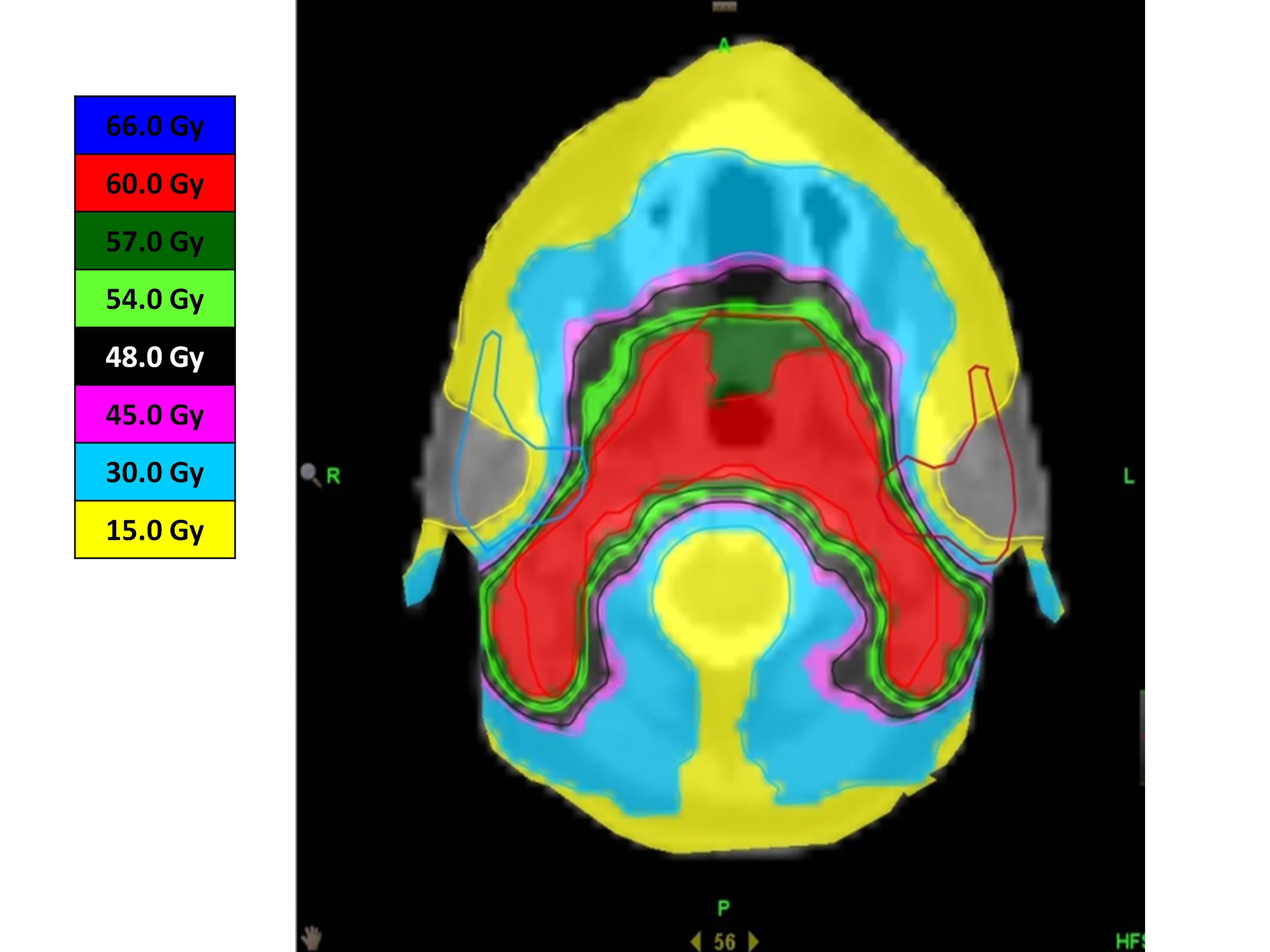

Supplement: Supplementary Figure 3 — Dose distribution of a head-and-neck cancer patient treated with helical TomoTherapy (66, 60, and 45 Gy in 30 fractions). Red contour for 60 Gy target, dark-red and cyan contours for right and left PGs respectively. Mean absorbed dose: 25.3, 25.9, 50.6, and 50.1 Gy for right and left PGs, right and left SGs respectively. [file Image_3.JPEG]

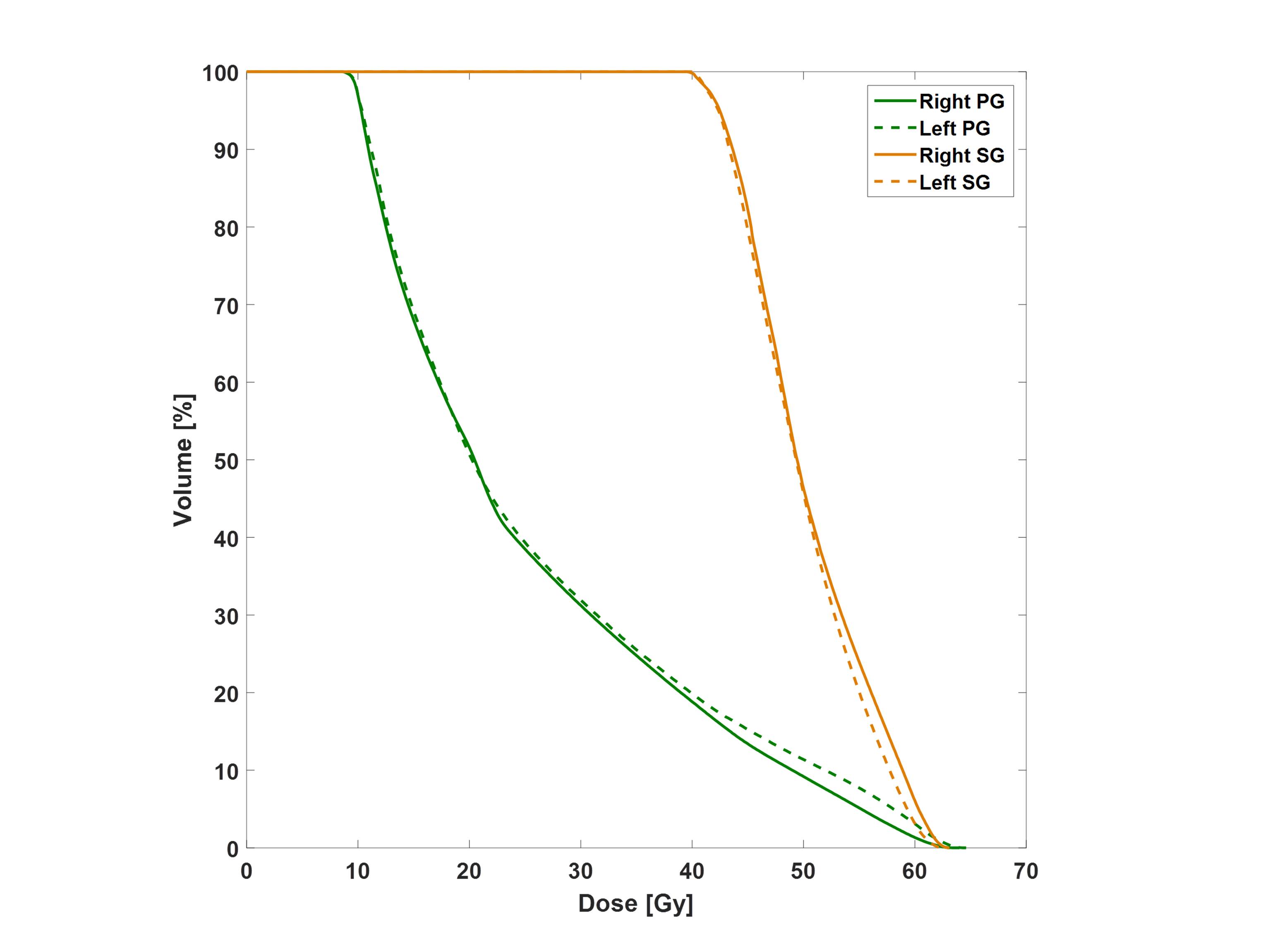

Supplement: Supplementary Figure 4 — Dose-volume histogram (DVH) of parotid and submandibular glands. [file Image_4.JPEG]
